# Internal jugular venous abnormalities in transient monocular blindness

**DOI:** 10.1186/1471-2377-13-94

**Published:** 2013-07-22

**Authors:** Chun-Yu Cheng, Feng-Chi Chang, A-Ching Chao, Chih-Ping Chung, Han-Hwa Hu

**Affiliations:** 1 Department of Neurology, Neurological Institute, Taipei -Veterans General Hospital, Taipei, Taiwan; 2 Department of Radiology, Taipei -Veterans General Hospital, Taipei, Taiwan; 3 Department of Neurology, Institute of Brain Science, National Yang-Ming University School of Medicine, Taipei, Taiwan; 4 Department of Radiology, Institute of Brain Science, National Yang-Ming University School of Medicine, Taipei, Taiwan; 5 Institute of Brain Science, National Yang-Ming University School of Medicine, Taipei, Taiwan; 6 Department of Neurology, Kaohsiung Medical University Chung-Ho Memorial Hospital, Kaohsiung, Taiwan; 7 Department of Neurology, Kaohsiung Medical University School of Medicine, Kaohsiung, Taiwan

**Keywords:** Transient monocular blindness, Internal jugular vein, Venous outflow abnormalities, Jugular venous reflux, Venous hypertension, Chronic cerebrospinal venous insufficiency

## Abstract

**Background:**

The etiology of transient monocular blindness (TMB) in patients without carotid stenosis has been linked to ocular venous hypertension, for their increased retrobulbar vascular resistance, sustained retinal venule dilatation and higher frequency of jugular venous reflux (JVR). This study aimed to elucidate whether there are anatomical abnormalities at internal jugular vein (IJV) in TMB patients that would contribute to impaired cerebral venous drainage and consequent ocular venous hypertension.

**Methods:**

Contrast-enhanced axial T1-weighted magnetic resonance imaging (MRI) was performed in 23 TMB patients who had no carotid stenosis and 23 age- and sex-matched controls. The veins were assessed at the upper IJV (at C1–3 level) and the middle IJV (at C3–5 level). Grading of IJV compression/stenosis was determined bilaterally as follows: 0 = normal round or ovoid appearance; 1 = mild flattening; 2 = moderate flattening; and 3 = severe flattening or not visualized.

**Results:**

There was significantly more moderate or severe IJV compression/stenosis in the TMB patients at the left upper IJV level and the bilateral middle IJV level. Defining venous compression/stenosis scores ≥ 2 as a significant cerebral venous outflow impairment, TMB patients were found to have higher frequency of significant venous outflow impairment at the upper IJV level (56.5% vs. 8.7%, p = 0.0005) and the middle IJV level (69.6% vs. 21.7%, p=0.0011).

**Conclusions:**

TMB Patients with the absence of carotid stenosis had higher frequency and greater severity of IJV compression/stenosis which could impair cerebral venous outflow. Our results provide evidence supporting that the cerebral venous outflow abnormality is one of the etiologies of TMB.

## Background

Transient monocular blindness (TMB) is defined as sudden, painless, and transient monocular vision loss. The most well recognized etiology of TMB is carotid atherothromboembolism [1,2]. However, the pathogenesis remains obscure in 10 to 56% of TMB patients [1-5]. In series of studies [5-9], we proved that jugular venous reflux (JVR) is involved in the pathogenesis of TMB. JVR implies an abnormal pressure gradient in the internal jugular vein (IJV), which might impede cerebral venous outflow and produce retrograde venous hypertension intracranially [10-12]. In TMB patients during the attack-free stage, we found increased vascular resistance in retrobulbar arteries in the absence of significant arterial lesions [6], which may result from impaired venous outflow. In patients with frequent TMB attacks of undetermined cause, the frequency of JVR was higher than the frequency observed in normal controls (20 to 40%) [8,11]. Furthermore, in a case–control study [7], TMB patients had a sustained wider retinal venule diameter, and this was especially true among TMB patients with JVR. These findings provided evidence that ocular venous hypertension due to cerebral venous outflow impairment may be one of the pathogeneses of TMB.

However, we still don’t know what causes these venous hemodynamic and structural abnormalities in TMB. In this study, we tried to test the hypothesis that TMB patients have greater frequency of anatomical abnormalities at IJV, which is the main cerebral venous outflow tract, compared with normal subjects. We used magnetic resonance imaging (MRI) analysis to study the morphology of IJV, so as to identify compression or stenosis of IJV in TMB patients.

## Methods

### Subjects

#### TMB patients and Age/gender-matched controls

We prospectively recruited patients diagnosed with TMB from consecutive outpatients of the Neurology Department of the Taipei Veterans General Hospital and from referrals for cerebrovascular survey by ophthalmologists or other physicians. All patients were examined by one neurologist and were questioned about the characteristics of their transient loss of vision using a standardized questionnaire. All of these TMB patients received color-coded duplex ultrasonography for cervical and intracranial vascular examination (Acuson; Sequoia, Mountain View, Calif., USA) performed by a single sonographer who was blinded to subjects’ clinical characteristics. The inclusion criteria were: (1) no identified underlying disease, such as carotid stenosis, heart disease, ophthalmologic diseases, or autoimmune diseases, and (2) ≥ 3 TMB attacks. The age- and sex- matched control group was selected from individuals receiving physical check-ups who had no carotid stenosis and no history of visual problems. Taipei Veterans General Hospital’s institutional review board approved the study proposal, and we obtained written informed consent from all subjects.

### MRI study

**Figure 1:**
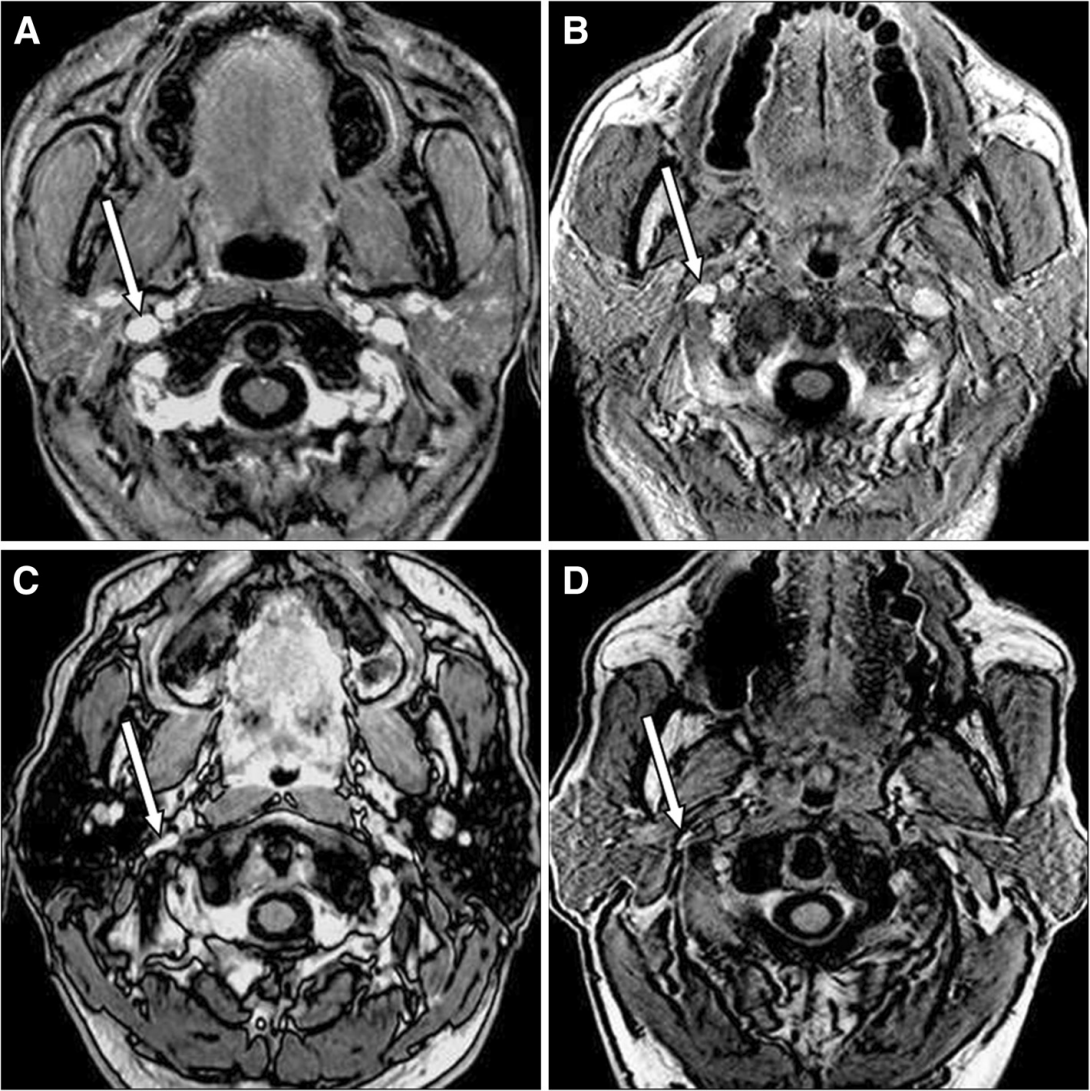
**MR imaging studies in a TMB patient with IJVs stenoses.** This is a 46 year-old lady with recurrent and transient monocular blindness over right eye. Contrast-enhanced, axial T1-weighted image **(A)** showed bilateral IJV stenoses (arrows). (right IJV: grade 1; left IJV: grade 3). MR angiogram **(B)** revealed decreased venous flow over left sided venous routes and focal narrowing over right sided IJV (arrow) while the arterial system was relatively normal, which was also shown on three-dimensional time-of-flight MR angiogram **(C)**.

We performed 1.5T MRI (Excite II; GE Medical Systems, Milwaukee, WI) on all participants. The imaging sequences were axial T1-weighted and contrast-enhanced axial T1-weighted images. The contrast-enhanced axial T1-weighted images extended from the skull to the thoracic inlet level in TMB patients (the same sequences of control individuals didn’t cover the lower IJV level and thoracic inlet), with the following parameters: repetition time (TR) = 8.6 milliseconds, echo time (TE) = 2.5 milliseconds, inversion time (TI) = 400 milliseconds, flip angle = 15°, slice thickness = 1.5 mm, field of view (FOV) = 24 cm, matrix = 320×256. Three-dimensional time-of-flight MR angiography (MRA) was applied for exclusion of patients with carotid stenosis and other obvious arterial sources that may result in the symptoms (Figure 1), with the parameters: repetition time (TR) = 30 milliseconds, echo time (TE) = 6.9 milliseconds, flip angle = 20°, slice thickness = 1.8 mm.

### Data analysis

**Figure 2:**
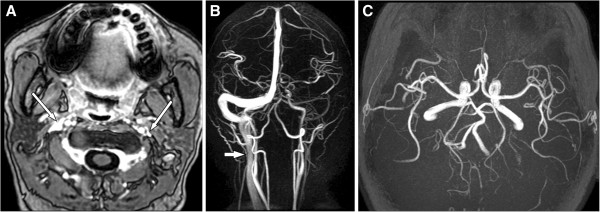
**Quantitative grading of IJV compression/stenosis.** Contrast-enhanced, axial T1-weighted images of quantitative grading of IJV compression/stenosis between 0 (none) and 3 (severe). Examples at the upper IJV level (C1-C3) demonstrate no stenosis **(A)** or mild **(B)**, moderate **(C)**, and severe **(D)** stenosis (arrows).

We assessed the veins’ morphologies at the upper IJV (at C1–3 level) and the middle IJV (at C3–5 level) using contrast enhanced axial T1-weighted MR images (Figure 2). We graded the venous compression/stenosis according to criteria by G. Zaharchuk et al. [13] as follows: grade 0 = normal round or ovoid appearance; grade 1 = mild flattening; grade 2 = moderate flattening; and grade 3 = severe flattening or not visualized. All subjects’ MRI readings were done by one neuro-radiologist and one neurologist. Both were well trained in neuroimaging reading and were blinded to the subjects’ clinical characteristics. Consensus meeting was conducted to discuss any problems or disagreements. The intraclass correlation coefficient for gradings was used to assess interrater agreement with an interrater reliability of 0.76.

### Statistical analysis

All values were expressed as mean ± standard deviation (SD) for continuous variables and number (percentage) for discrete variables. A Chi-square test was performed to test the difference between TMB patients and controls. An adjusted p value of < 0.025 was considered significant for multiple comparisons.

## Results

The clinical characteristics and IJV compression/stenosis grading of TMB patients and age/gender-matched normal individuals are summarized in Table 1.

**Table 1:** Clinical characteristics and grading of IJV stenosis in TMB patients and age/gender-matched control subjects

	**TMB (N=23)**	**Controls (N=23)**	**p value**
Age	49±18	49±18	
Sex (F/M)	10/13	10/13	
**Middle IJV stenosis**			
Left side			0.0036
None	4 (17%)	15 (65%)	
Grade 1	10 (43%)	7 (30%)	
Grade 2	5 (22%)	1 (4%)	
Grade 3	4 (17%)	0	
Right side			0.0167
None	10 (43%)	19 (83%)	
Grade 1	3 (13%)	3 (13%)	
Grade 2	8 (35%)	1 (4%)	
Grade 3	2 (9%)	0	
**Upper IJV stenosis**			
Left side			0.0173
None	7 (30%)	11 (48%)	
Grade 1	3 (13%)	9 (39%)	
Grade 2	8 (35%)	2 (9%)	
Grade 3	5 (22%)	1 (4%)	
Right side			0.8415
None	11 (48%)	13 (56%)	
Grade 1	6 (26%)	6 (26%)	
Grade 2	4 (17%)	2 (9%)	
Grade 3	2 (9%)	2 (9%)	

There was significantly more moderate or severe IJV compression/stenosis in TMB patients at the left upper IJV level and bilateral middle IJV level (Table 1). Severe flattening or not visualized venous caliber (grade 3) at either side of the upper and middle IJV was found in 10 TMB patients and in 3 controls (43.5% vs. 13.0%, p = 0.022). At either side of the upper and middle IJV, the prevalence of venous compression/stenosis rated ≥ 1 was significantly higher in patients than in controls (78.3% vs. 56.5.0%, p = 0.026). Defining moderate and severe compression/stenosis with IJV (score ≥ 2) as a significant cerebral venous outflow impairment, 13 TMB patients in contrast to only 2 controls had a significant venous outflow impairment at the upper IJV level (56.5% vs. 8.7%, p = 0.0005), and 16 patients in contrast to only 5 controls had a significant venous outflow impairment at the middle IJV level (69.6% vs. 21.7%, p = 0.0011).

## Discussion

TMB Patients with the absence of carotid stenosis had higher frequency and greater severity of IJV compression/stenosis which could impair cerebral venous outflow and consequent ocular venous hypertension. IJV outflow abnormalities may be one of the etiologies of TMB. TMB is a rare disease and the average annual incidence rate was reported to be around 7.4/100,000 population [3,14]. While atherothrombotic origin is the most clearly demonstrated mechanism of TMB, 10% of the TMB patients were left without clinical evidence of organic arterial or cardiac diseases [1,3,15,16]. In this study, we focused on TMB patients without carotid stenosis or other arterial sources that may be contributed to the symptoms. Therefore, our small sample size of patient and matched-control groups are valid to demonstrate the relationship between TMB of unknown etiologies and IJV abnormalities. The novelty of our study is that we are the first to use contrast-enhanced T1 MR imaging to evaluate IJV in TMB patients, and the results will open a new window exploring the pathophysiologies of TMB.

Human and animal studies examining cerebral venous occlusion have shown that an elevated venous pressure would result in a dilatation or/and venous reflux in upstream venule beds [17-19]. In our previous studies [7,8], TMB patients, even during the attack-free stage, had altered ocular hemodynamics, including flow reversal in the superior ophthalmic vein [8], increased resistance in retrobulbar arteries [6] and dilatation of the retinal venule [7]. Higher frequency and greater severity of IJV compression/stenosis at both the upper and middle levels in TMB, demonstrated by the present study, may be the etiology of these ocular hemodynamic abnormalities. Compression/stenosis of IJV would lead to decreased venous flow volume, and consequently less degree of venous pressure in IJV. On the contrary, the pressure of the downstream venous routes, such as brachiocephalic vein, is relatively higher. This may explain the higher frequency of venous reflux in IJV found in transient global amnesia and TMB patients [7,20]. The reflux was proposed to be related to the abnormal venous pressure gradient resulted from the lower venous pressure and structural abnormalities in IJV.

Recently, severe IJV stenosis and other cerebral venous drainage abnormalities (chronic cerebrospinal venous insufficiency; CCSVI) have been linked to multiple sclerosis (MS) [21-23]. Retinal pathology and abnormal ocular venous morphologies were more commonly found in MS patients with IJV occlusion [24]. Extracranial venoplasty for patients with CCSVI was reported to result in significant clinical improvement [25]. Moreover, quantitative flow measurements using MR phase contrast imaging showed reduced blood flow through the IJVs in MS patients with IJV stenoses [26,27]. Our preliminary data showed a trend that flow volume decrease detected on sonography was correlated with severity of IJV stenotic grading (upper IJV level) on MRI in TMB patients. Although, one of our limitations in the study was the unavailability to perform the promising quantitative MR measurements, the sonographic findings supported previous studies [26,27] that IJV stenosis could make influences on the hymodynamic changes in IJV. Data from controls are needed for comparison.

There are studies demonstrating etiologies of IJV stenosis, such as external compression from bony structure [28,29] or adjacent muscle [30]. Truncular venous malformation involving the venous system of the head and neck, an embryological defect, has also been reported as one of the causes of CCSVI [31]. We did found higher frequency of BCV compression in TMB patients. Since lacking of imaging survey from controls, more studies are needed for evaluating the lower IJV and also venous routes down to the thorax. Then, comparison and further judgments can be drawn. Vascular, infectious and inflammatory factors were shown to be associated with cerebral venous outflow impairment in another study [32]. These are all possible underlying causes, individually or in combination, of IJV stenosis in our TMB patients. Further studies are needed to identify the underlying mechanisms of IJV stenosis in these patients.

## Conclusions

This is the first study that demonstrates IJV compression/stenosis in TMB patients. With evidences provided by our previous studies, IJV outflow impairment leading to cerebral/ocular venous drainage impairment and consequent ocular venous hypertension may be one of the etiologies of TMB.

## Abbreviations

TMB: Transient monocular blindness; JVR: Jugular venous reflux; IJV: Internal jugular vein; MRI: Magnetic resonance imaging; 3D TOF MRA: Three-dimensional time-of-flight MR angiography; BCV: Brachiocephalic vein; CCSVI: Chronic cerebrospinal venous insufficiency; MS: Multiple sclerosis.

## Competing interests

The authors report no conflict of interest concerning the materials or methods used in this study or the findings specified in this paper. All authors declare no financial or non-financial competing interests.

## Authors’ contributions

CYC: contributions to conception and design, acquisition of data, analysis and interpretation of data, and drafting the manuscript. FCC: contributions to conception and design, and revising the manuscript. ACC: contributions to conception and design. CPC: contributions to conception and design, analysis and interpretation of data, and revising the manuscript. HHH: Contributions to conception and design, acquisition of data, analysis and interpretation of data, and revising the manuscript. All authors read and approved the final manuscript.

## Pre-publication history

The pre-publication history for this paper can be accessed here:

http://www.biomedcentral.com/1471-2377/13/94/prepub
